# Calling Where It Counts: Subordinate Pied Babblers Target the Audience of Their Vocal Advertisements

**DOI:** 10.1371/journal.pone.0130795

**Published:** 2015-07-15

**Authors:** David J. Humphries, Fiona M. Finch, Matthew B. V. Bell, Amanda R. Ridley

**Affiliations:** 1 Department of Biological Sciences, Macquarie University, Sydney, NSW, Australia; 2 Institute of Evolutionary Biology, School of Biological Sciences, University of Edinburgh, Edinburgh, EH9 3JT, United Kingdom; 3 DST/NRF Centre of Excellence at the Percy FitzPatrick Institute, University of Cape Town, Cape Town, Western Cape, South Africa; 4 Pied Babbler Research Project, Kuruman River Reserve, Van Zylsrus, South Africa; 5 Centre for Evolutionary Biology, School of Animal Biology, University of Western Australia, Perth, WA, Australia; Université Lyon 1, FRANCE

## Abstract

For territorial group-living species, opportunities to reproduce on the natal territory can be limited by a number of factors including the availability of resources within a territory, access to unrelated individuals, and monopolies on reproduction by dominant group members. Individuals looking to reproduce are therefore faced with the options of either waiting for a breeding opportunity to arise in the natal territory, or searching for reproductive opportunities in non-natal groups. In the cooperatively breeding Southern pied babbler, *Turdoides bicolor*, most individuals who achieve reproductive success do so through taking up dominant breeding positions within non-natal groups. For subordinate pied babblers therefore, searching for breeding opportunities in non-natal groups is of primary importance as this represents the major route to reproductive success. However, prospecting (where individuals leave the group to search for reproductive opportunities within other groups) is costly and individuals rapidly lose weight when not part of a group. Here we demonstrate that subordinate pied babblers adopt an alternative strategy for mate attraction by vocal advertisement from within their natal territories. We show that subordinates focus their calling efforts on the edges of their territory, and specifically near boundaries with neighbouring groups that have potential breeding partners (unrelated individuals of the opposite sex). In contrast to prospecting, calling individuals showed no body mass loss associated with this behaviour, suggesting that calling from within the group may provide a ‘cheap’ advertisement strategy. Additionally, we show that subordinates use information regarding the composition of neighbouring groups to target the greatest number of potential mating partners.

## Introduction

For species that live in family groups, opportunities to reproduce on the natal territory are limited by both the availability of resources within a territory, and access to unrelated individuals [1–3]. An additional limitation arises when reproduction within the group is monopolised by a single dominant pair [4–6]. For subordinates within these groups, opportunities to reproduce are restricted to either waiting for a breeding opportunity to become available within the natal territory, or searching for breeding opportunities in the surrounding area [7,8].

While subordinate group members of some cooperative species do take over breeding positions within the natal group, these occurrences are usually rare, owing to the high relatedness of group members and the limited number of opportunities available (particularly in long lived species; [7,8–13]). It is therefore important that subordinates invest in searching for reproductive opportunities outside of the natal group where there are several pathways to reproductive success, including: (1) taking over a breeding position within a non-natal group (either by taking up vacant breeding positions or by taking breeding positions by force)[7]; (2) attempting to found a new group [7]; or (3) engaging in prospecting behaviour to achieve extra-group paternity [14,15]. In all three of these strategies subordinates must signal their intentions to individuals that live outside of the natal territory.

Signalling breeding availability and searching for breeding opportunities beyond the natal territory can be costly [16]. Prospecting, where subordinates of reproductive age leave their natal groups for short periods to search for breeding opportunities in non-natal groups, is a common strategy among group-living animals [17–21]. However, prospectors are constrained by the energetic costs of being away from the group [22,23]. These costs are thought to result from the increase in movement and vigilance behaviours, decline in foraging activity, risk of attack from territory holders, and the associated stress among individuals that are outside of a group [21–24]. An alternative strategy to prospecting is to advertise breeding availability from within the natal territory. This is a little explored alternative, yet it carries the potential for individuals to remain within their social group and continue to receive the benefits of group living (such as a reduced investment in personal vigilance and reduced predation risk [22,25,26]).

For subordinates wishing to advertise their breeding availability, some neighbouring groups may hold greater reproductive opportunities than others, with variation in both the number, relatedness and quality of potential partners [27]. In cooperatively breeding birds, the combination of delayed dispersal and short dispersal distances can often lead to a high probability of encountering close kin in the local neighbourhood [28]. Inbreeding can be detrimental to reproductive success [29,30], consequently, we might expect individuals advertising for mates to concentrate their efforts on unrelated neighbouring groups. An additional factor for group-living animals is that neighbouring groups may vary in the number of opposite sex individuals they contain. This can result in variation in the number of individuals that can be reached from an advertisement, depending on the location where it is produced. We may therefore expect advertisement calls to occur at locations that reach the greatest number of potential mating partners. Whether subordinates are strategic in their search for breeding opportunities and adopt strategies to maximise their exposure to potential breeding partners is currently poorly understood in cooperatively breeding species.

We investigated the advertising strategies used by subordinate Southern pied babblers, *Turdoides bicolor*, who begin to produce loud-calls from within their natal territories when they reach reproductive age (> 1 year old). Pied babblers are a medium sized (75–95g) passerine endemic to the Kalahari, living in social groups of 2–15 individuals [31]. Breeding within the social group is monopolised by a dominant pair [6], and subordinate individuals will only achieve dominance within their natal territory if they can inherit vacant breeding positions without inbreeding [7,13]. Prospecting in pied babblers is costly [22], and long-term floating is rarely observed (80.0% of prospectors return to their natal group within 30 days; A. Ridley, unpublished data). In some species, prospecting can achieve immediate reproductive success [14,32], however in the pied babbler, genetic studies have revealed no evidence of extra-group parentage of young [6]. Consequently, prospecting is unlikely to represent a significant route to short-term reproductive success [6]. Here we set out to determine whether subordinate individuals adopt strategies to target the audience of their vocal advertisements by a) calling on the edges of their territory, b) focusing their calling efforts near to unrelated groups, and c) focusing their calling efforts near to groups with the greatest number of unrelated, opposite sex, adult individuals. We also assess whether calling behaviour carries observable costs and therefore whether calling from within the natal group provides an energetically costly route to advertising breeding availability.

## Methods

Subordinate advertisements were recorded from a population of pied babblers located at the Kuruman River Reserve, in the Southern Kalahari, South Africa (26°57’S 21°49’E) (see [33] for more details about the study site). The pied babbler is neither endangered nor protected and all work was carried out with ethical clearance provided by the University of Cape Town, approved under ethics number R2012/2006/V15/AR. This study was carried out on private land. The reserve manager David Gaynor should be contacted for future permissions. We would like to thank Prof Tim Clutton-Brock, Prof Marta Manser and the Kuruman Reserve Trust for access to their land. We thank the Northern Cape Conservation Authority for research permits.

The population of pied babblers is colour-ringed for identification and has been under observation since 2003. We have detailed life history information for each of the individuals and groups within the population, including information on dominance hierarchies and the movement of individuals between groups. We recorded the loud-calling behaviour of subordinate individuals across two breeding seasons, between September 2010—April 2011, and September 2011—April 2012. Throughout these periods, data was recorded twice a day, with a morning session from dawn (mean observation time ± SD 140.60±53.72 minutes per group visited), and an afternoon session till dusk (mean observation time ± SD 82.64±50.46 minutes per group visited). The population is habituated to close observation (within five metres) and full group compositions are recorded in every data collection session. Pied babblers move and forage as a cohesive group and it is possible to continuously monitor the behaviour of all individuals in the group reliably. Pied babblers produce eight acoustically distinct types of loud-call, all of which are predominantly given by the dominant members of the group [34]. From acoustic recording data of loud-calls where both the calls, caller identities and behavioural contexts were collected, loud calls were observed being given by subordinate members in just 23.85% of cases (249 of 1044 audio recorded loud-calls). This bias towards loud-calling from dominant individuals occurs despite the number of subordinates outweighing the number of dominants 2.3 to 1 in the population. Loud-calling behaviour can occur in a wide variety of social contexts [34], for instance, during group chorusing, whilst moving fledglings, and to relocate the group should an individual become separated. One of the most prevalent times when loud-calling is observed is when a dominant male loses his mate through death or divorce. The dominant male will give frequent loud-calls until a new female joins the group, at which point the loud-calling rate will decline significantly (A Ridley, unpublished data). Based on these observations, we assume that loud-calling is associated with mate advertisement. Prospecting individuals also use repeated loud-calling during prospecting events, supporting the notion that loud-calls can function for self-advertisement (D. Humphries, personal observation). When loud-calling occurs in the contexts described above (during group chorusing, whilst moving fledglings, and while moving between foraging areas), the associated behaviour of the individual makes the causality of calling clear to a trained observer. However, here we focused on the rare instances when subordinates gave solo loud-calls when no behavioural context could be observed instigating the calling behaviour. These loud-calls are prominent vocal displays, typically given at ~70dB (when recorded from 5m with a sound pressure meter; D. Humphries personal observation) from a high vantage point and can be heard up to 500m from the calling individual and are easily detected and recorded. We deemed solo loud-calls given without perceivable initiating circumstances to be self-advertisements. Pied babblers can breed after their first year [7], and unsolicited loud-calling behaviour is observed almost exclusively by adult subordinate individuals (mean ± SD 1027±321 days since hatching; range 327–1536), with only 1.9% of cases observed by individuals under one year of age (adults are defined as at least one year post-hatching). Dispersal in the pied babbler does not appear to be sex-biased [35], and subordinate loud-calling behaviour occurs in both sexes. Of the cases where subordinates were observed calling, only 26.10% had no clear social context, and could be defined as self-advertisement (65 out of 249 recorded vocalisations from 33 individuals belonging to 13 social groups).

### Where do subordinates call within a territory?

#### Sampling self-advertisement loud call locations

Each time a subordinate was observed giving loud-call advertisements, the location of the calling behaviour was recorded to a handheld GPS (accuracy <10m). We limited our analysis to individuals where we had recorded the locations of at least ten loud-calls (mean±SD 26.40 ± 10.00; range 10–38) across a breeding year (September through to August). Loud-calls could be given repeatedly from the same location and were classified as independent calls when there had been at least one second of non-calling behaviour between calls (n = 264 loud calls recorded, from 104 different locations). We recorded calling locations from seven individuals from six different social groups. Three individuals produced at least 10 calls in two seasons providing a total of n = 10. While repeated use of individuals and groups introduces psudeoreplication into our data, the scarcity of data and the novelty of this research topic make all samples of interest.

#### Sampling group movements

In addition to recording the location of loud-calling behaviour, the movements of the whole social group were recorded every 15 minutes to a handheld GPS during every observation session. Pied babblers move slowly around their territories, typically covering approx 1km in a 2–3 hour observation session. Territory sizes were established from 300 GPS points collected across a breeding year. 300 points represents a minimum of 60 hours of observation for each group. Territory sizes were calculated using the ‘adaptive sphere-of-influence local convex hull’ (a-LoCoH; [36]). A-LoCoH was performed in R 2.15.1 (R Development Core Team 2008) using the ‘adehabitat’ package [37]. We exported territories at three different density isopleths (contour lines indicating the smallest area that encompasses 50%, 75%, and 95% of the GPS location points for each group), from R in to ArcGIS 9.3.1 (ESRI, 2009) for analysis. We investigated whether subordinate calling behaviour followed patterns of group movement. For example, whether half of all loud-calling behaviour was observed within the 50% density isopleths, 25% between the 50 and 75% density isopleths, and 20% within the area between the 75 and 95% isopleths. We explored both the number of loud calls given and the number of calling locations in each area of the territory, If subordinate calling behaviour did not follow patterns of group movement, it would suggest that subordinates are favouring particular locations (e.g. the border vs the centre of the territory) for calling. We compared observed versus expected (calculated as the total number of calls given in each location multiplied by the proportion expected e.g. 0.5 within the 50% isopleth, 0.25 within the 75% isopleth etc) calling patterns using a Chi-squared test. All statistical tests were carried out in R.

### Does relatedness to neighbouring groups affect where subordinates advertise?

**Fig 1:**
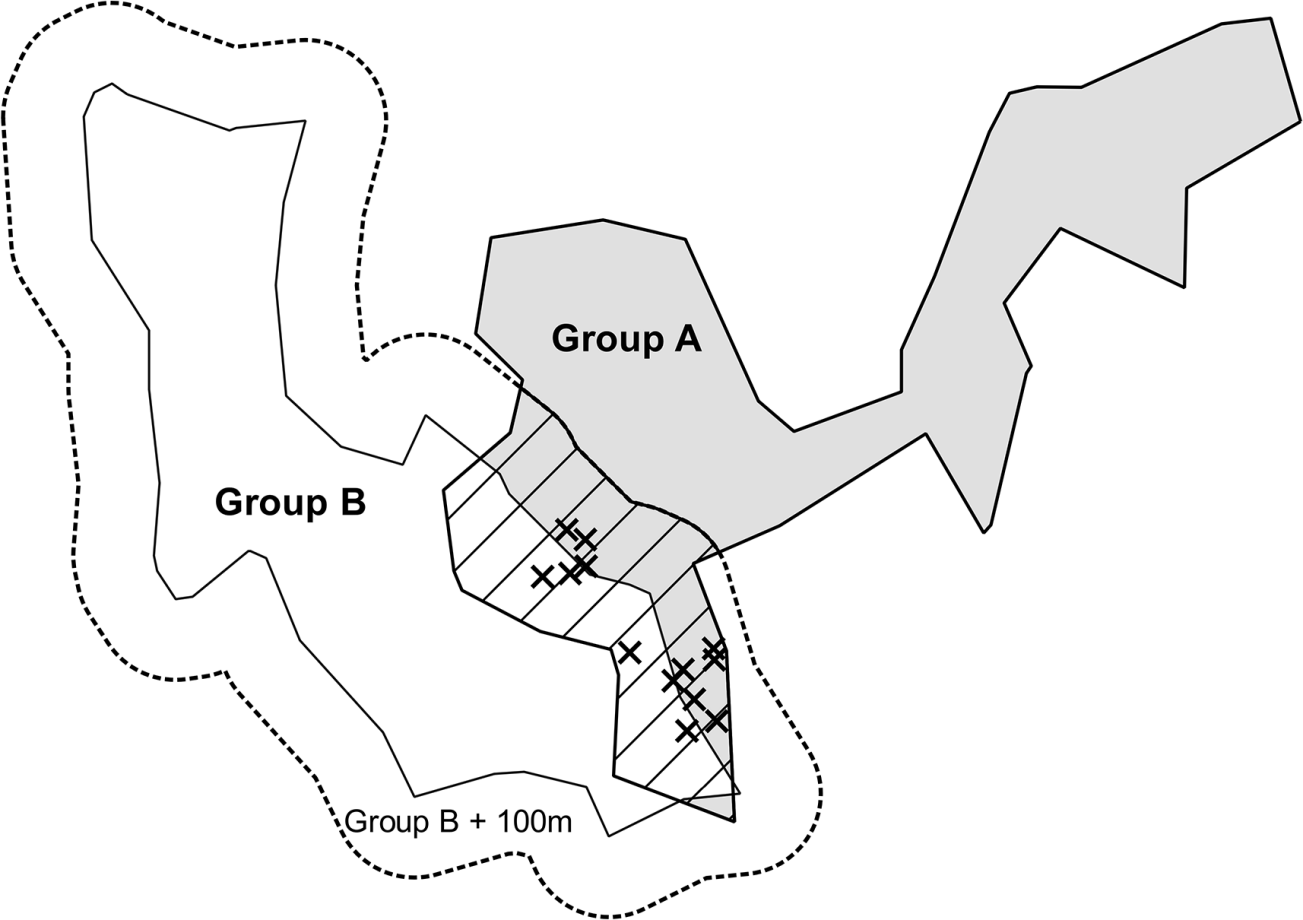
A schematic demonstrating how we established proximity to neighbouring territories. A 100m buffer zone was created around the 95% density isopleths of neighbouring groups. We then established the number of loud-calls (shown here as crosses) that fell within 100m of the neighbouring territories (inclusion area shown here as a hashed area). Image created in ArcGIS 9.3.1.

We examined the distribution of individual calling behaviour within each territory to see if calling behaviour was focused towards boundaries with neighbouring groups containing unrelated, potential breeding partners. Using the ‘buffer zone’ tool in the ‘Hawths tools’ extension (Beyer, H. L., 2004. Hawth's Analysis Tools for ArcGIS. Available at http://www.spatialecology.com/htools) we created a 100m zone around the 95% density isopleths of neighbouring territories. We then established a) the number of self-advertisement calls given by each focal subordinate and b) the number of calling locations occurring within 100m of a neighbouring territory for each focal subordinate (see Fig 1 for a schematic; 100m represents approximately 5% of a territory diameter on average). The number of calls observed was assessed relative to what would be expected if subordinate calling behaviour was evenly distributed within a territory. The expected number of calls was calculated as the total number of calls given by a focal individual, divided by the area of the 95% density isopleth (in hectares), multiplied by the area that was in a 100m proximity to a neighbouring group. We classified groups as related when at least one dominant of the neighbouring group was a close relative (r = 0.25 or closer) to the calling individual. In pied babblers, parentage can be reliably assigned from behavioural observations of breeding behaviour and activity at the nest [6]. To establish relatedness between individuals, pedigrees were developed from behavioural observations of parentage. These relationships were confirmed using genetic analysis (see [13] for details). We compared observed versus expected calling patterns for both the number of calls and the number of calling locations at related (n = 8 cases where the calling individual had at least one related neighbouring group) and unrelated boundaries with neighbouring groups (n = 9) using a Chi squared test. Additionally, in seven cases the caller had both a related and unrelated neighbouring group. We compared the number of calls and the number of calling locations on related and unrelated borders using a paired t-test.

We investigated whether the number of calls given by an individual in proximity to a neighbouring group was affected by the number of adult individuals within the neighbouring group. This was to investigate whether loud-calling effort was influenced by the size of the potential audience. There were seven cases where the caller had more than one neighbouring group and for these seven we explored a) the number of calls per hectare and b) the number of calling locations per hectare given in proximity to their largest neighbouring group compared to their smallest neighbouring group. We analysed the observed rates using a paired t-test carried out in R. The mean difference between the largest and smallest neighbouring group sizes for focal individuals in this study was 1.73±1.39 individuals (mean±SD). We also ran an analysis looking at whether the number of unrelated, opposite sex adult individuals within neighbouring groups affected calling behaviour. This was to explore whether a specific audience (potential breeding partners) was being targeted through vocal advertising. We tested whether the number of calls per hectare was higher in proximity to groups that contained the most unrelated, opposite sex adult individuals, relative to the neighbouring group that contained the least. We also explored calling behaviour on the borders containing the most adult, unrelated neighbours that were the same sex as the caller and compared this to the border containing the least. Pied babblers are sexually monomorphic and require genetic sexing from blood samples collected during ringing (following the method described in [38]).The mean difference between the maximum and minimum number of unrelated, opposite sex adult individuals within neighbouring groups was 2.32±1.57 individuals (mean±SD). We tested differences in observed calling behaviour from the same individual near different groups using a related samples paired t-test.

### Costs of calling

The population is habituated to the use of weighing scales and will stand on a top-pan scale (Ohaus CS200; accuracy ± 0.1g) in exchange for a small reward (small amounts of egg and mealworm). Assessing patterns of daily weight gain provides a useful mechanism for calculating whether activities carry substantial costs, and have previously been used to assess the costs of floating [22] and extreme heat [39] in pied babblers. To investigate whether advertising from within the social group is a costly mechanism of advertising for mates, we compared daily weight change on days when we observed at least six advertisement calling bouts from an individual (mean number of advertisement calls 11.5, range 6–30) and again on days where no advertisement calling bouts were observed. Weight change from eight individuals in seven different groups were recorded (where weight change is defined as the difference in grams between the weight at the start of the observation session compared to the weight at the end. Comparable weight sessions occurred within two weeks of each other to minimise seasonal effects on weight gain (mean±SD 4.88±3.52 days apart; range 2–11 days). We compared within-individual differences in daily weight gain using a paired t-test. All graphs presented were produced in R.

## Results

### Does calling behaviour follow group movement?

**Fig 2:**
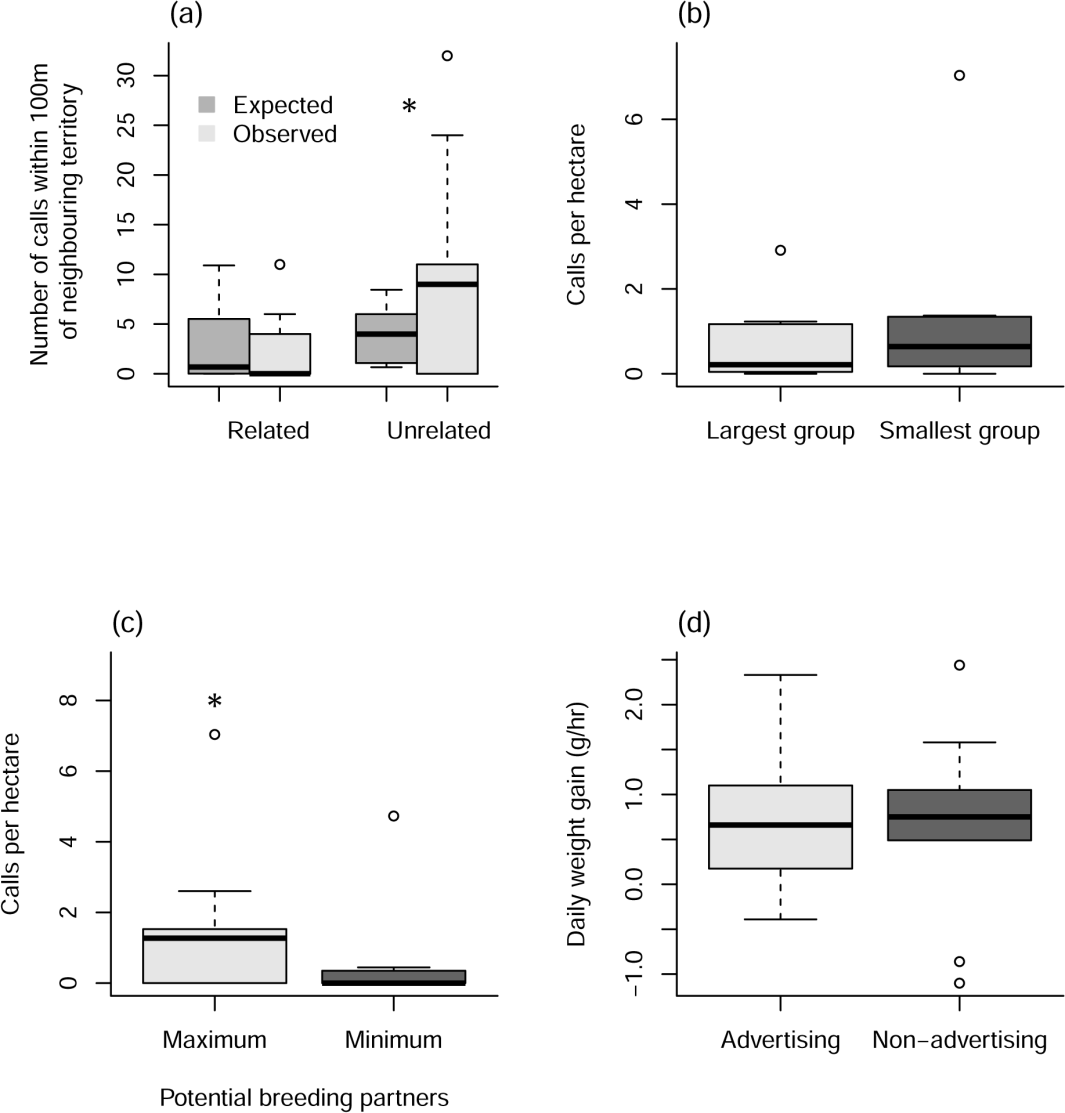
The observed and expected number of loud-calls given by each individual within the 50% density isopleths, between the 50 and 75% isopleths, and between the 75–95% (n = 10).

Calling behaviour differed significantly from patterns of group movement with both the number of calls (Χ^2^ = 34.710, df = 2, P = <0.001), and the number of calling locations (Χ^2^ = 6.768, df = 2, P = 0.004), occurring more frequently on the outer reaches of a territory (between the 75 and 95% density isopleths) than we would have expected if calling behaviour followed group movement patterns (Fig 2; S1 Fig).

### Is calling behaviour affected by the relatedness of neighbouring groups?

**Fig 3:**
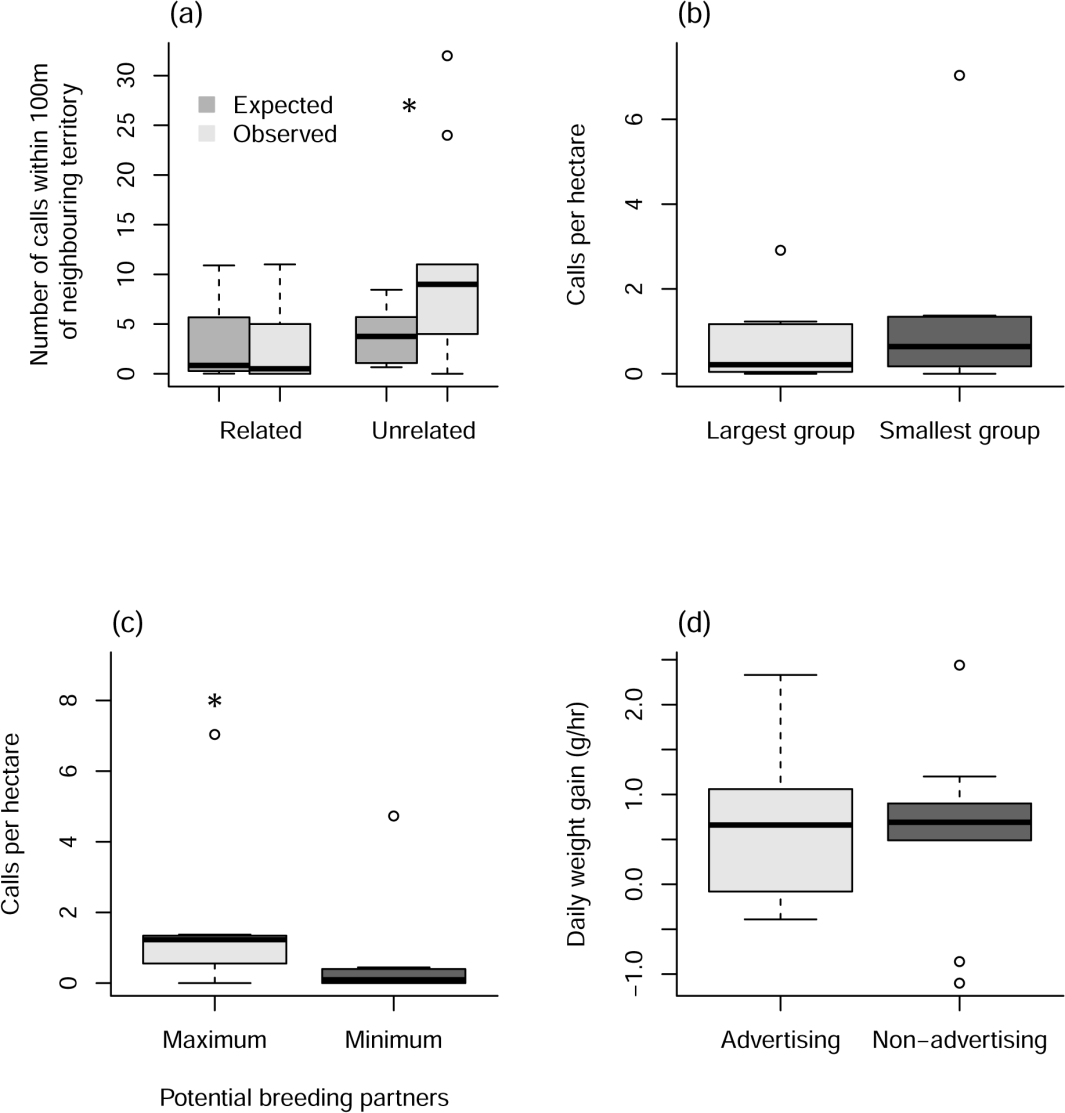
(a) The expected and observed number of loud-calls per hectare occurring within 100m of the territories of both related (n = 8) and unrelated neighbouring groups (n = 9). (b) The number of calls per hectare occurring in proximity to an individual’s largest and smallest neighbouring group, in terms of the number of adult individuals they contain (n = 7). (c) The number of calls per hectare occurring in proximity to an individual’s largest and smallest neighbouring group, in terms of the number of unrelated, opposite sex adult individuals they contain (n = 7). (d) The daily weight gain of individuals both when we observed at least six loud-calling bouts, and when no loud-calling behaviour was witnessed (n = 10).

The relatedness of neighbouring groups affected subordinate calling behaviour, with both the number of calls (Χ^2^ = 11.895, df = 1, P = <0.001) and the number of calling locations (Χ^2^ = 6.768, df = 1, P = 0.009), occurring more frequently in proximity to unrelated groups than would be expected if calling behaviour occurred evenly throughout the territory (Fig 3A; S2 Fig). In seven cases the caller had both related and unrelated neighbouring groups for which calling data could be compared, and in six of those the caller produced more calls per hectare in the boundaries of unrelated groups than related boundaries. However, this effect was not significant for either the number of calls (paired t-test, t = -1.380, df = 6, P = 0.217) or the number of calling locations (paired t-test, t = -1.801, df = 6, P = 0.122). In the one case where more calls per hectare were produced on a related boundary, the calls occurred in an area that also overlapped with the boundary of an unrelated neighbouring group.

### Is calling focused near groups with the greatest number of unrelated, opposite sex individuals?

Subordinates were not found to focus their loud-calling on the boundaries with their largest neighbouring group, with both the number of calls (Fig 3B; paired t-test, t = 1.156, df = 6, P = 0.291) and the number of calling locations (S3 Fig; paired t-test, t = 1.438, df = 6, P = 0.201) not significantly different when compared to the calling behaviour at the border with the smallest group. However, when we looked at whether subordinates targeted groups that had the highest number of unrelated, opposite sex adult individuals, we found that they called more in proximity to their neighbouring group that contained the highest number of potential breeding partners, giving both more calls per hectare (Fig 3C; paired t-test, t = 3.544, df = 6, P = 0.012) and calling in more locations per hectare (S4 Fig; paired t-test, t = 3.805, df = 6, P = 0.022) when compared to their neighbouring group that contained the fewest potential breeding partners. When we compared calling behaviour on borders with neighbouring groups containing the maximum number of unrelated adults that were the same sex as the caller, it was not significantly more than calling behaviour on the border containing the fewest (paired t-test, t = -0.291, df = 6, P = 0.781), neither was the number of calling locations (paired t-test, t = 3.805, df = 6, P = 0.022). This suggests that they are maximising their exposure to a specific target audience with their advertisements.

### Cost of calling

We observed no difference in daily weight gain on days when advertisements were observed when compared to non-advertising days (Fig 3C; S5 Fig; t-test, t = 0.645, df = 7, P = 0.540). This suggests that the cost of advertising from within the natal territory is minimal.

## Discussion

Our findings that subordinate loud-calling behaviour is concentrated on the edges of territories, and specifically near to groups containing a number of unrelated, opposite sex individuals suggests that unsolicited loud-calling by subordinates functions for mate advertisement. Importantly, it also suggests that pied babblers are capable of discriminating kinship and the *number* of potential mates within neighbouring groups, and can utilise this information to maximise the audience of their calling efforts.

The ability to discriminate kinship has previously been demonstrated in avian species, which utilise vocal [40–43], visual [44–46], and olfactory signals [47,48] to recognise kin. By avoiding kin as mating partners, an individual can limit the potentially damaging effects of inbreeding depression among resultant offspring, and therefore improve reproductive success [30,49]. Genetic analysis of parentage in the pied babbler has previously found that breeding between familiar relatives is rare and that they are therefore likely to have a mechanism of recognising familiar kin which they utilise to avoid inbreeding [13]. Our observations further support the idea that pied babblers can recognise kin and behaviourally discriminate relatives in their environment.

One of the contexts in which loud-calling behaviour is observed is when the caller is in search of a mating partner, however, the calls are multi-functional and can be given in a wide range of contexts [34]. It is possible that the calling patterns we have observed are serving another function for the caller. For instance if the calls function for territorial defence, they may still occur at a higher frequency on boundaries with unrelated neighbouring groups. Reduced aggression and a greater tolerance to related neighbours has been observed in a range of taxa including fish [50], birds [51], and mammals [52–55]. However, in the pied babbler territorial defence is usually undertaken as a group with all adult group members chorusing together [56].

Our findings indicate that subordinates are maximising the potential of their loud-calling behaviour by using information regarding the composition of neighbouring groups. This information is likely to be obtained through several mechanisms. Firstly, information may be exchanged during inter-group interactions. Baboons, *Papio cynocephalus*, use inter-group encounters to assess the number of opposite sex individuals within neighbouring groups [57]. Pied babblers frequently engage in ritualised inter-group interactions and have many opportunities for information exchange [56]. During inter-group interactions, pied babblers often utilise sex-specific loud-calling behaviour [34], which may provide a mechanism for assessing the number of opposite-sex individuals in neighbouring groups. Secondly, information regarding the composition of neighbouring groups may be obtained from prospecting bouts, with information-gathering considered one of the primary functions of prospecting behaviour [58,59]. Or thirdly, information may be gained through eavesdropping on neighbours [60]. Great tits, *Parus major*, are able to assess the quality of neighbouring males by eavesdropping on their calling behaviour [61]. Eavesdropping may similarly provide a way of obtaining information about the composition of neighbouring groups in pied babblers.

Despite our observations that pied babblers are targeting a specific audience with their vocal displays, the benefits of this behaviour remain unclear. One possibility is that calling serves to initiate encounters with neighbouring groups, facilitating the exchange of information about reproductive opportunities. Subordinate members of both meerkat, *Suricata suricatta* [62], and banded mongoose, *Mungos mungo* [63] groups are observed leading the social group into encounters with neighbouring groups (although in these species extra-pair paternity and subordinate reproduction mean that immediate reproductive success may be gained through such encounters [62,63]). Regular information exchange between neighbouring groups may also be important for dispersal success [64]. In Brown Jays, *Cyanocorax morio*, for example, dispersal occurs most frequently between neighbouring groups where rates of interaction are high [58]. Loud-calling behaviour may therefore serve a dual function of both advertising the caller, and encouraging information exchange through inter-group interactions with neighbouring groups.

When we compared daily weight gain on days where we observed subordinate loud-calling to weight gain on days when no calling behaviour was observed, we found no significant difference. This is in contrast to prospecting events, where individuals lose body condition when living outside of a social group [22]. When individuals are away from the social group, they invest more time in vigilance behaviours and experience reduced foraging success [22]. By advertising from within the social group, pied babblers can continue to experience the benefits of living within the social group (such as shared vigilance and better predator detection [22,25,26]), which may explain why we did not observe any difference in their daily weight gain. Advertising from within the natal territory is therefore an energetically cheap route to advertising breeding availability when compared to prospecting.

We have observed that calling behaviour is non-random, and focused on the borders of territories with potential mating partners. However, the strength of our conclusions may be limited by the inability to explore key aspects such as non-independence in the calling data. Calls occurring repeatedly from the same location, or observed within a single observation session are not independent data points. This problem is less pronounced in the locational data where all the trends were significant. Exploring calling behaviour requires detailed life history information on the caller, the territory of its social group as well as the territories of neighbouring groups and knowledge of the relatedness between neighbouring social groups. These criteria have limited the available sample size for this study, which has restricted the analyses we can perform. Future research would benefit from being able to statistically control for non-independence of observations.

Here we have described how subordinate pied babblers, in addition to prospecting for breeding opportunities in the wider area [7], also adopt a strategy of vocalising to neighbouring groups from within their natal territory. This strategy is maximised by using information regarding the composition of neighbouring groups to target an audience of potential breeding partners. Importantly, subordinate loud-calling is not just given to any neighbouring group, nor focused towards the largest groups, but subordinate pied babblers are specifically targeting unrelated groups that contain a number of opposite sex individuals. Our findings provide fresh insight into how subordinates within cooperatively breeding-societies, that are constrained in their opportunities to breed on the natal territory, appear to use information about the composition of neighbouring groups to inform the location of their vocal displays to target an audience of potential breeding partners.

## Supporting Information

S1 Fig Calling rates observed in each density isopleth. (a) Boxplots for the observed and expected number of loud-calls given by each individual within the 50% density isopleths, between the 50 and 75% isopleths, and between the 75–95%. (b) Raw data for the observed and expected number of loud-calls given by each individual within the 50% density isopleths, between the 50 and 75% isopleths, and between the 75–95%. (c) Boxplots for the observed and expected number of loud-calling locations of each individual within the 50% density isopleths, between the 50 and 75% isopleths, and between the 75–95%. (d) Raw data for the observed and expected number of loud-calling locations of each individual within the 50% density isopleths, between the 50 and 75% isopleths, and between the 75–95% (n = 10).
(TIF)

S2 Fig Calling rates observed on borders with related and unrelated neighbours. (a) Box-plots of the expected and observed number of loud-calls per hectare occurring within 100m of the territories of both related (n = 8) and unrelated neighbouring groups (n = 9). (b) Raw data of the expected and observed number of loud-calls per hectare occurring within 100m of the territories of both related (n = 8) and unrelated neighbouring groups (n = 9). (c) Box-plots of the expected and observed number of loud-calling locations per hectare occurring within 100m of the territories of both related (n = 8) and unrelated neighbouring groups (n = 9). (d) Raw data of the expected and observed number of loud-calling locations per hectare occurring within 100m of the territories of both related (n = 8) and unrelated neighbouring groups (n = 9).
(TIF)

S3 Fig Calling rates observed on the borders of the largest and smallest neighbouring groups. (a) Box-plots of the number of calls per hectare occurring in proximity to an individual’s largest and smallest neighbouring group, in terms of the number of adult individuals they contain (n = 7). (b) Raw data of the number of calls per hectare occurring in proximity to an individual’s largest and smallest neighbouring group, in terms of the number of adult individuals they contain (n = 7). (c) Box-plots of the number of calling locations per hectare occurring in proximity to an individual’s largest and smallest neighbouring group, in terms of the number of adult individuals they contain (n = 7). (d) Raw data of the number of calling locations per hectare occurring in proximity to an individual’s largest and smallest neighbouring group, in terms of the number of adult individuals they contain (n = 7).
(TIF)

S4 Fig Calling rates observed on the borders of neighbouring groups with the maximum and minimum number of potential breeding partners. (a) Box-plots of the number of calls per hectare occurring in proximity to an individual’s largest and smallest neighbouring group, in terms of the number of unrelated, opposite sex adult individuals they contain (n = 7). (b) Raw data of the number of calls per hectare occurring in proximity to an individual’s largest and smallest neighbouring group, in terms of the number of unrelated, opposite sex adult individuals they contain (n = 7). (c) Box-plots of the number of calling locations per hectare occurring in proximity to an individual’s largest and smallest neighbouring group, in terms of the number of unrelated, opposite sex adult individuals they contain (n = 7). (d) Raw data of the number of calling locations per hectare occurring in proximity to an individual’s largest and smallest neighbouring group, in terms of the number of unrelated, opposite sex adult individuals they contain (n = 7). of the expected and observed number of loud-calling locations per hectare occurring within 100m of the territories of both related (n = 8) and unrelated neighbouring groups (n = 9).
(TIF)

S5 Fig Daily weight gain on calling and non-calling days. (a) Box-plots of the daily weight gain of individuals both when we observed at least six loud-calling bouts, and when no loud-calling behaviour was witnessed (n = 10). (b) Raw data of the daily weight gain of individuals both when we observed at least six loud-calling bouts, and when no loud-calling behaviour was witnessed (n = 10)
(TIF)

S1 Source File Diagrams showing the distribution of calling behaviour for each of the individuals included in the study for each season. (XLSX)
