# Supplementary figures and images for: Calling Where It Counts: Subordinate Pied Babblers Target the Audience of Their Vocal Advertisements
Source: PLoS One. 2015 Jul 15;10(7):e0130795. doi: 10.1371/journal.pone.0130795 (PMC4503734; doi:10.1371/journal.pone.0130795)

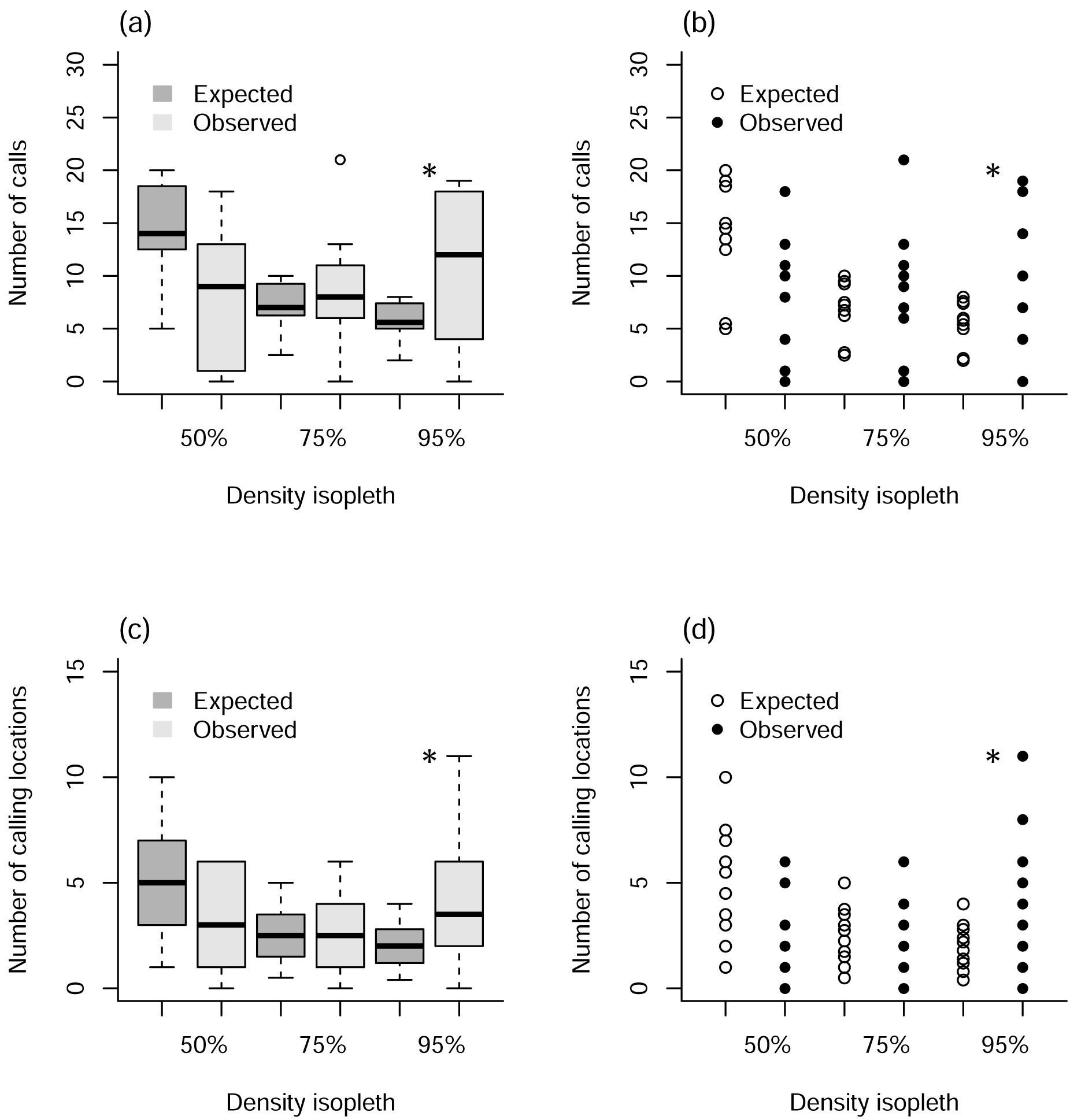

Supplement: S1 Fig — (a) Boxplots for the observed and expected number of loud-calls given by each individual within the 50% density isopleths, between the 50 and 75% isopleths, and between the 75–95%. (b) Raw data for the observed and expected number of loud-calls given by each individual within the 50% density isopleths, between the 50 and 75% isopleths, and between the 75–95%. (c) Boxplots for the observed and expected number of loud-calling locations of each individual within the 50% density isopleths, between the 50 and 75% isopleths, and between the 75–95%. (d) Raw data for the observed and expected number of loud-calling locations of each individual within the 50% density isopleths, between the 50 and 75% isopleths, and between the 75–95% (n = 10). (TIF) [file pone.0130795.s001.tif]

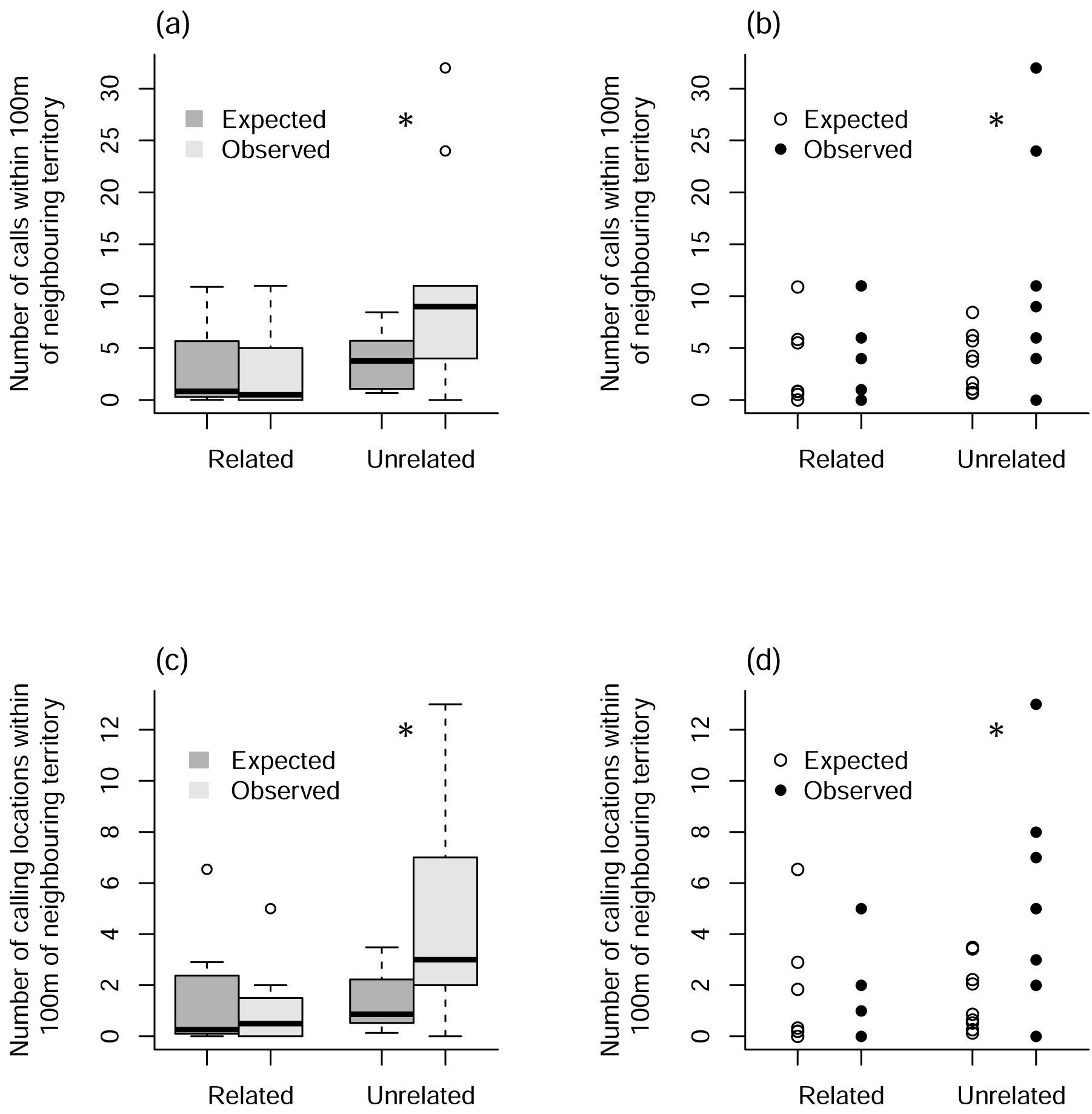

Supplement: S2 Fig — (a) Box-plots of the expected and observed number of loud-calls per hectare occurring within 100m of the territories of both related (n = 8) and unrelated neighbouring groups (n = 9). (b) Raw data of the expected and observed number of loud-calls per hectare occurring within 100m of the territories of both related (n = 8) and unrelated neighbouring groups (n = 9). (c) Box-plots of the expected and observed number of loud-calling locations per hectare occurring within 100m of the territories of both related (n = 8) and unrelated neighbouring groups (n = 9). (d) Raw data of the expected and observed number of loud-calling locations per hectare occurring within 100m of the territories of both related (n = 8) and unrelated neighbouring groups (n = 9). (TIF) [file pone.0130795.s002.tif]

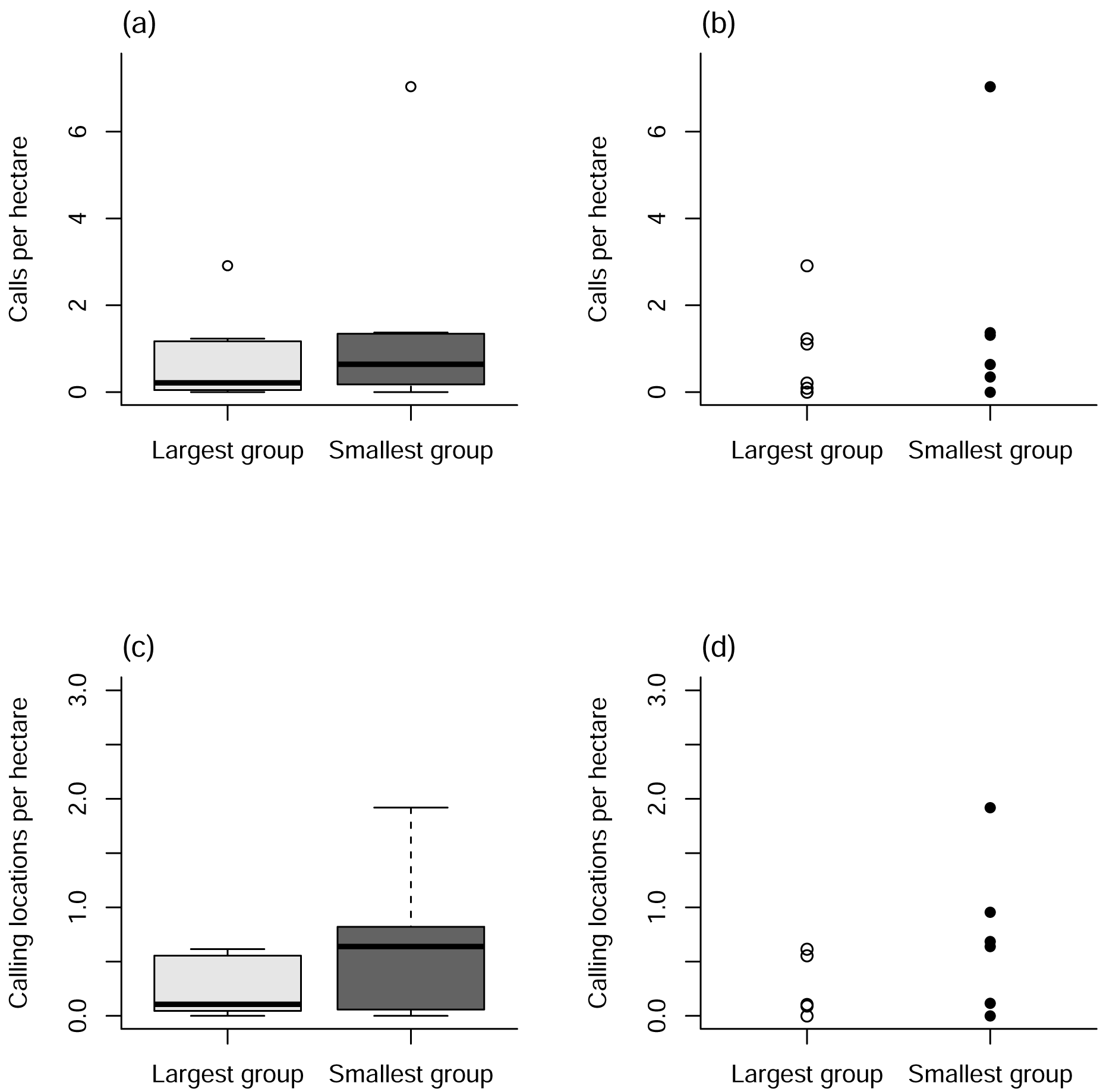

Supplement: S3 Fig — (a) Box-plots of the number of calls per hectare occurring in proximity to an individual’s largest and smallest neighbouring group, in terms of the number of adult individuals they contain (n = 7). (b) Raw data of the number of calls per hectare occurring in proximity to an individual’s largest and smallest neighbouring group, in terms of the number of adult individuals they contain (n = 7). (c) Box-plots of the number of calling locations per hectare occurring in proximity to an individual’s largest and smallest neighbouring group, in terms of the number of adult individuals they contain (n = 7). (d) Raw data of the number of calling locations per hectare occurring in proximity to an individual’s largest and smallest neighbouring group, in terms of the number of adult individuals they contain (n = 7). (TIF) [file pone.0130795.s003.tif]

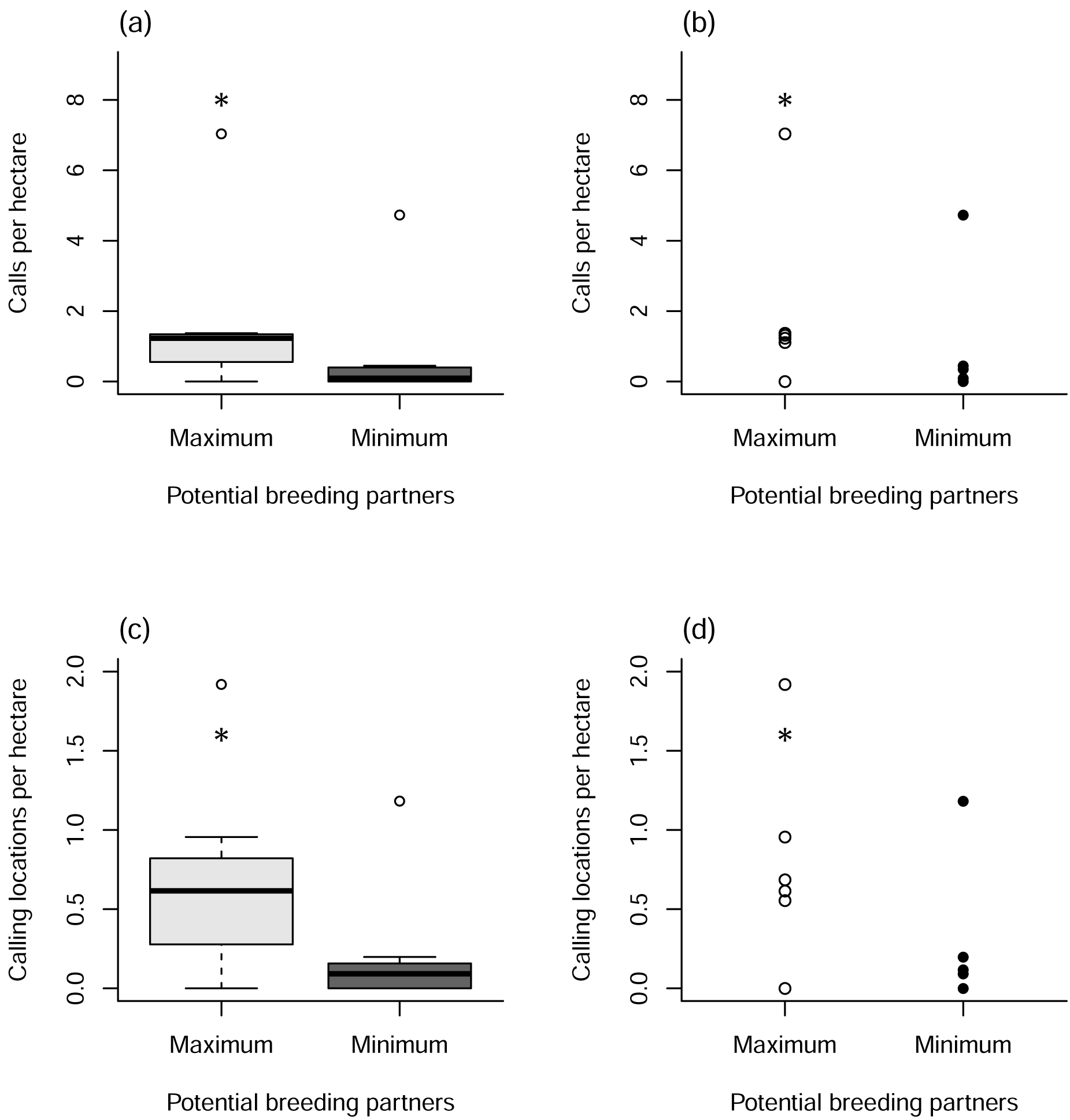

Supplement: S4 Fig — (a) Box-plots of the number of calls per hectare occurring in proximity to an individual’s largest and smallest neighbouring group, in terms of the number of unrelated, opposite sex adult individuals they contain (n = 7). (b) Raw data of the number of calls per hectare occurring in proximity to an individual’s largest and smallest neighbouring group, in terms of the number of unrelated, opposite sex adult individuals they contain (n = 7). (c) Box-plots of the number of calling locations per hectare occurring in proximity to an individual’s largest and smallest neighbouring group, in terms of the number of unrelated, opposite sex adult individuals they contain (n = 7). (d) Raw data of the number of calling locations per hectare occurring in proximity to an individual’s largest and smallest neighbouring group, in terms of the number of unrelated, opposite sex adult individuals they contain (n = 7). of the expected and observed number of loud-calling locations per hectare occurring within 100m of the territories of both related (n = 8) and unrelated neighbouring groups (n = 9). (TIF) [file pone.0130795.s004.tif]

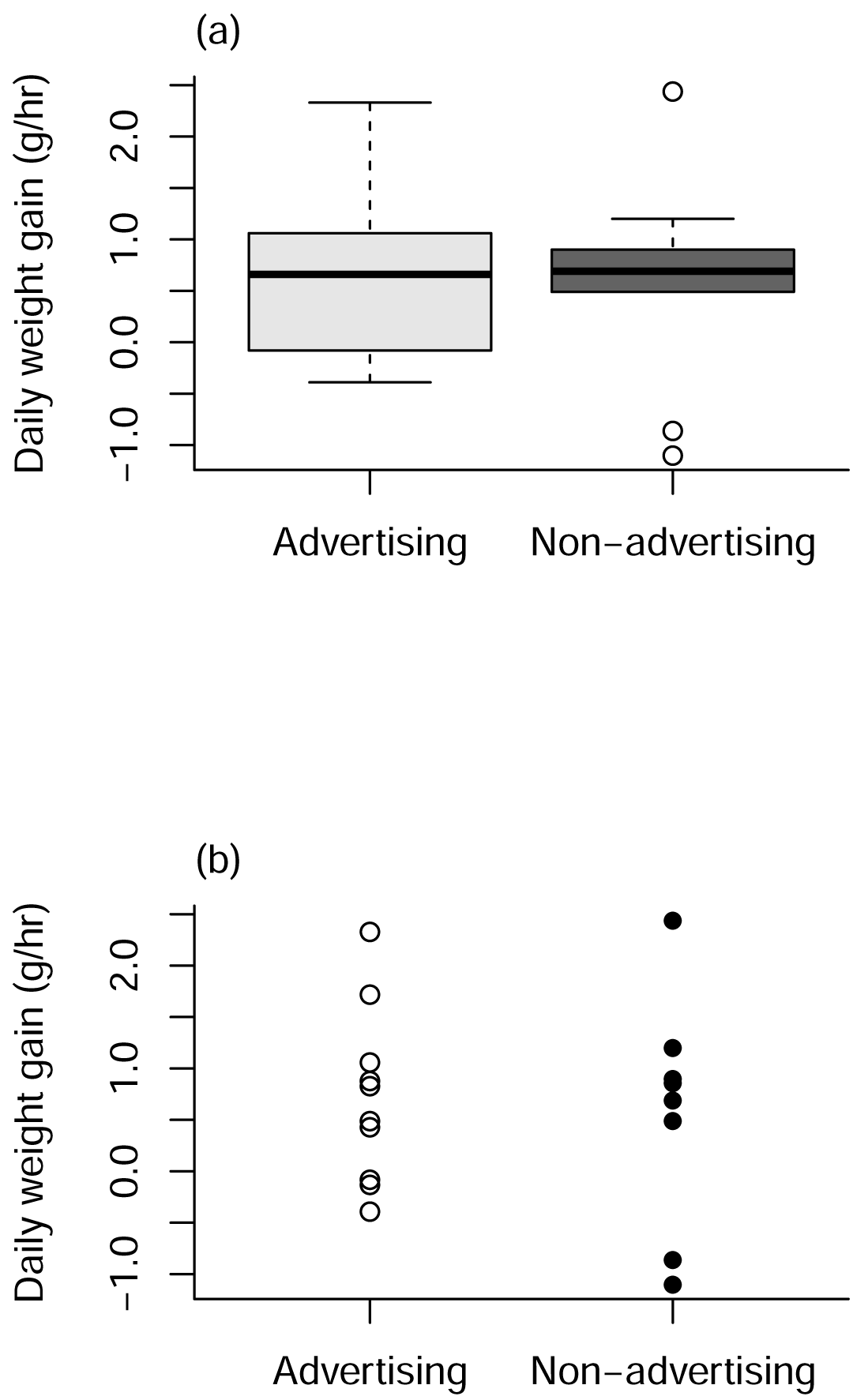

Supplement: S5 Fig — (a) Box-plots of the daily weight gain of individuals both when we observed at least six loud-calling bouts, and when no loud-calling behaviour was witnessed (n = 10). (b) Raw data of the daily weight gain of individuals both when we observed at least six loud-calling bouts, and when no loud-calling behaviour was witnessed (n = 10) (TIF) [file pone.0130795.s005.tif]
